# Tenascin-C induces inflammatory mediators and matrix degradation in osteoarthritic cartilage

**DOI:** 10.1186/1471-2474-12-164

**Published:** 2011-07-15

**Authors:** Lisha Patel, Weiyong Sun, Sonya S Glasson, Elisabeth A Morris, Carl R Flannery, Priya S Chockalingam

**Affiliations:** 1 Tissue Repair, BioTherapeutics Research & Development, Pfizer, Cambridge, MA, USA

## Abstract

**Background:**

Tenascin-C (TN-C) is an extracellular matrix glycoprotein that is involved in tissue injury and repair processes. We analyzed TN-C expression in normal and osteoarthritic (OA) human cartilage, and evaluated its capacity to induce inflammatory and catabolic mediators in chondrocytes *in vitro*. The effect of TN-C on proteoglycan loss from articular cartilage in culture was also assessed.

**Methods:**

TN-C in culture media, cartilage extracts, and synovial fluid of human and animal joints was quantified using a sandwich ELISA and/or analyzed by Western immunoblotting. mRNA expression of TN-C and aggrecanases were analyzed by Taqman assays. Human and bovine primary chondrocytes and/or explant culture systems were utilized to study TN-C induced inflammatory or catabolic mediators and proteoglycan loss. Total proteoglycan and aggrecanase -generated ARG-aggrecan fragments were quantified in human and rat synovial fluids by ELISA.

**Results:**

TN-C protein and mRNA expression were significantly upregulated in OA cartilage with a concomitant elevation of TN-C levels in the synovial fluid of OA patients. IL-1 enhanced TN-C expression in articular cartilage. Addition of TN-C induced IL-6, PGE_2_, and nitrate release and upregulated ADAMTS4 mRNA in cultured primary human and bovine chondrocytes. TN-C treatment resulted in an increased loss of proteoglycan from cartilage explants in culture. A correlation was observed between TN-C and aggrecanase generated ARG-aggrecan fragment levels in the synovial fluid of human OA joints and in the lavage of rat joints that underwent surgical induction of OA.

**Conclusions:**

TN-C expression in the knee cartilage and TN-C levels measured in the synovial fluid are significantly enhanced in OA patients. Our findings suggest that the elevated levels of TN-C could induce inflammatory mediators and promote matrix degradation in OA joints.

## Background

Tenascin-C (TN-C) is a modular, multifunctional extracellular matrix (ECM) glycoprotein that is associated with tissue injury and repair. It was discovered originally in gliomas, muscle tissue and in the nervous system, and called by different names: myotendinous antigen, glial/mesenchymal ECM protein, cytotactin, J1 220/200, neuronectin and hexabrachion [1]. It was later found in the osteotendinous junction and superficial layers of articular cartilage [2,3]. The structure of TN-C comprises an amino-terminal oligomerization domain consisting of heptad repeats, multiple epidermal growth factor (EGF)-like repeats, fibronectin type III repeats (FN-III) and a carboxyl-terminal fibrinogen-like globular domain. It forms a hexameric 1.5 million Da form through the formation of disulfide links N-terminal to the triple-coiled coil region of two trimers [4].

TN-C interacts with a variety of ECM molecules and cell surface receptors, thus affecting tissue architecture, tissue resilience and cell responses. It plays a major role in cell adhesion, migration, proliferation, and cellular signaling through induction of pro-inflammatory cytokines [5]. TN-C is abundantly expressed during embryogenesis and organogenesis. Its expression is highly restricted in healthy adult tissues, but reappears in the process of wound healing, regeneration, or neoplastic events [6,7]. TN-C is associated with the development of articular cartilage, but decreases markedly during maturation of chondrocytes [8,9], and almost disappears in adult cartilage [10,11]. In diseased conditions including osteoarthritis (OA) and rheumatoid arthritis (RA), TN-C is highly expressed in both cartilage and synovium [10-13]. A correlation between TN-C levels in synovial fluid and degree of cartilage degradation [14] or radiographic progression of knee OA [15] has been shown.

The proinflammatory cytokine, IL-1 plays a significant role in joint pathology, and its actions can occur through TLR4 (Toll-like receptor-4) activation [16]. Bobacz *et al*. confirmed the expression of TLR4 in human articular chondrocytes at both the mRNA and the protein level [17]. Lipopolysaccharides (LPS) induce catabolic effects in cartilage matrix [18]; LPS-induced activation of TLR4 in articular chondrocytes has been shown to decrease matrix biosynthesis [17]. TN-C was recently identified as an endogenous DAMP (damage-associated molecular pattern) activating TLR4 in inflammatory diseases [19]. TN-C is also reported to induce cytokine and metalloprotease (MMP) synthesis in murine synovial fibroblasts *via *activation of α9 integrins [20]. Intra-articular injection of TN-C promoted joint inflammation *in-vivo *in mice, and mice that do not express TN-C showed rapid resolution of acute joint inflammation and are protected from erosive arthritis induced by immunization and intra-articular injection of methylated BSA [19].

The objective of the current study was to compare cartilage mRNA and protein levels of TN-C under normal and OA conditions, and determine the effect of IL-1 on TN-C expression in articular cartilage. We also evaluated the role of TN-C in inducing inflammatory mediators and proteoglycan degradation in articular cartilage. TN-C levels were correlated with proteoglycan levels in the synovial fluid samples of OA patients; and the pattern of TN-C release as compared to aggrecanase-generated ARG-aggrecan fragment release into synovial fluid was followed in a rat model of OA.

## Methods

### Human cartilage extract and RNA preparation

Specimens of non-OA cartilage obtained from human donors with consent had no history of joint disorders; they were received within 36 hours following autopsy (National Disease Research Interchange, Philadelphia, PA), examined macroscopically and smooth intact surfaces without OA-like lesions were harvested for protein extraction and RNA preparation. The average age of the 7 non-OA donors was 43 years with an age range of 38-58 years. Specimens of OA cartilage with visible lesions were obtained with consent from patients undergoing knee replacement surgery at New England Baptist Hospital, and harvested within a few hours of surgery. The average age of the 7 OA cartilage donors was 68 years with an age range of 50-82 years. This study was performed under the approval of Pfizer's Institutional Human Ethics Committee. Cartilage slices harvested under sterile conditions were cut into explants (6-8 mm width), rinsed three times in PBS, and flash frozen. Cartilage (1 g each) was pulverized in a Spex Certiprep freezer mill Model 6750 (15 Hz; 2 × 1 min with 1 min pause between cycles) under liquid nitrogen for protein extraction and RNA preparation. RNA was prepared from pulverized cartilage as described [21]. For protein extraction, the powdered cartilage was immediately suspended in 10 ml of 4 M guanidine HCl, 50 mM sodium acetate pH 5.8 containing protease inhibitor cocktail (Calbiochem, Gibbstown, NJ) and extracted for 48 hours at 4°C on a rotator. The mixture was then centrifuged at 3,000 rpm for 10 min and the supernatant dialyzed against 20 mM Tris-HCl, pH 8.2 (with protease inhibitors) overnight at 4°C. OA and non-OA cartilage extracts (5 μg protein equivalent) were deglycosylated with 0.15 U/ml chondroitinase ABC (Sigma, St Louis, MO), 0.15 U/ml Keratanase I and 0.0075 U/ml Keratanase II (Seikagaku America, Falmouth, MA) at 37°C for 3 hours. The samples were separated on a 3-8% Tris-Acetate gel (Invitrogen, Carlsbad, CA), transferred to nitrocellulose membrane and probed with anti-human Tenascin-C (EGF-like domain) antibody 4F10TT (Immuno-Biological Laboratories Co., Ltd. Japan) at 1:100 dilution followed by incubation in anti-mouse IgG (H&L) conjugated to alkaline phosphatase (Promega, Madison, WI) at 1:3000 dilution. Detection of reactive bands was performed with NBT/BCIP substrate (Promega). Purified human TN-C protein (Millipore, Billerica, MA) was used as a positive control in the Western blot analysis. The blots were also probed with secondary antibody alone to confirm specificity of detection.

### Endotoxin removal

Purified human TN-C protein (Millipore) from human glioma cell line U251 was used in the *in vitro *experiments. Endotoxin levels in the TN-C protein samples were measured using the Endosafe Portable Test System (Endosafe-PTS) in a cartridge, PTS 201 with a sensitivity range of 10-0.1 EU/ml (Charles River Laboratories, Wilmington, MA). The protein was taken through an endotoxin removal process using detoxigel endotoxin removal columns (ThermoFisher Scientific, Waltham, MA) following manufacturer's protocol. The endotoxin levels were measured again in the TN-C preparation using the cartridge, PTS 2005 (5-0.05 EU/ml range sensitivity) and the Endosafe-PTS after endotoxin removal.

### Primary chondrocyte cultures

Bovine and human primary chondrocytes were prepared under sterile conditions by pronase and collagenase treatments followed by filtration and centrifugation as previously described [22]. Cells were washed, resuspended in DMEM-F12 (Gibco, Carlsbad, CA), 10% FBS (Sigma, St. Louis, MO), 1% antimycotic-antibiotic solution (Sigma), and counted on a hemocytometer. Cell viability was determined by trypan blue dye exclusion, cell viability was found to be >95%. Cells were plated at 1 million/well in a 24 well tissue culture plate (1 ml/well) and maintained at 37°C. The cells were serum starved overnight once they were confluent (in 3-4 days), and washed with serum free media before induction. LPS from E. coli R515 (Re) (Axxora, San Diego, CA) at 0 to 1000 ng/ml or TN-C protein at 0 to 10 μg/ml was added and incubated for 48 hours at 37°C to study dose-dependent induction of primary chondrocytes. Heat killed TN-C that was heated at 100°C for 30 min, and LPS preincubated for 1 hour with polymyxin-B (PMB; Sigma, St. Louis, MO) served as negative controls for TN-C and LPS treatment, respectively. TN-C at 10 μg/ml preincubated with 3 μg/ml PMB was also tested to confirm that the induction effects observed with TN-C were not endotoxin related. TAK242, a specific TLR4 inhibitor [23], was synthesized at Pfizer. For TAK242 treatment, the cells were pretreated with inhibitor alone for 2 hours prior to induction with 1000 ng/ml LPS or 10 μg/ml TN-C in the presence of inhibitor. The media was removed after 48 hours of induction and analyzed in IL-6, nitrate, and PGE_2 _assays. Cells treated with 1000 ng/ml LPS, 10 μg/ml TN-C or 5 ng/ml IL-1β with or without TAK242 for 48 hours were washed in PBS, and lysed in lysis buffer for RNA preparation using RNAeasy kit (Qiagen, Valencia, CA) following the manufacturer's protocol.

### Cartilage explant cultures

Articular cartilage explant discs were harvested under sterile conditions from young bovine metacarpal phalangeal joints (Research 87, Hopkinton, MA). Briefly, full-thickness plugs were punched using a 8 mm cork borer and cartilage discs were generated by slicing 1 mm thick sections from the articular surface of the plugs. Discs were rinsed in PBS and subsequently cultured in medium. The medium consisted of Dulbecco's Modified Eagle's medium (JRH Biosciences, Lenexa, KS), 50 μg/ml ascorbic acid (Wako, Osaka, Japan), 10 mM HEPES (Mediatech, Herndon, VA), 2 mM L-glutamine (Mediatech), antibiotic-antimycotic solution (Sigma). Discs were cultured for 5 days with one media change in a 37°C and 5% CO_2 _environment to equilibrate the tissue prior to treatment. Following equilibration, 3 discs (~100 mg/well) were weighed and placed in 24-well tissue culture plate in 1 ml medium with or without 1 or 10 ng/ml of IL-1α (Sigma, St Louis, MO) for 48 hours for the first study. The media was tested for TN-C levels, and RNA prepared from cartilage discs for TN-C taqman analysis. For the second study, explants were treated with 5 ng/ml IL-1α, 10 μg/ml TN-C, or 1000 ng/ml LPS with or without TAK242 (0.01, 0.1, or 1 μM). For TAK242 effects, explants were pre-treated with the inhibitor for 2 hours prior to induction in the presence of inhibitor. The media was removed for the analysis of proteoglycan release after 48 hours of induction.

### Synovial fluid samples

Neat human knee joint synovial fluids from patients with end stage osteoarthritis (OA; n = 8: 4 males and 4 females; 50-83 years old) were obtained from NEBH, and synovial fluids from knee-healthy reference subjects (Ref; n = 8: 5 males and 3 females; 45-56 years old) were from NDRI or Northland labs with patient consent. The OA group included 7 synovial fluids of the same donors from whom cartilage samples were used for TN-C protein and mRNA expression. Representative OA and reference synovial fluids (15 μl each) from the above set were treated with 10 U of hyaluronidase (Sigma) at RT overnight and subjected to Western blot analysis with anti-human Tenascin-C (EGF-like domain) antibody 4F10TT as described above for cartilage extracts. The blots were probed with secondary antibody alone to confirm specificity of detection.

Male Lewis rats weighing approximately 300 grams were obtained from Charles River Laboratories (Wilmington, MA). The rats underwent medial meniscal surgery in the right knee to induce joint instability leading to cartilage degeneration as described [24]. The animals were euthanized at different times after surgery (4 days, 1 wk, 2 wks, and 3 wks; n = 10 animals/time point). Synovial fluid lavages and serum were collected. Five naïve animals per time point were also included. Serum and synovial fluid lavage urea levels in each rat were used to correct TN-C, proteoglycan, and ARG-aggrecan values for dilution. This study was performed under the approval of Pfizer's Institutional Animal Care and Use Committee.

### Biochemical assays

TN-C was measured in cartilage extracts, conditioned media, and synovial fluid samples using the TN-C Large ELISA kit (Immuno-Biological Laboratories Co., Ltd. Japan). The ELISA uses anti-TN-C 19C4MS monoclonal antibody against the FNIII-C domain for capture, and HRP conjugated anti-TN-C 4F10TT mouse monoclonal antibody against the EGF domain for detection. 4F10TT binds an epitope from the EGF domain and recognizes both the small and large TN-C variants. 19C4MS binds an epitope of the FNIII-C domain and recognizes large variants. The characteristics of these antibodies have been described elsewhere [15,25]. TN-C standard in the kit was run at 0-24 ng/ml for a standard curve. Samples were appropriately diluted in PBS and assayed in the TN-C ELISA using manufacturer's protocol. TN-C standard or human synovial fluid samples incubated in PBS- or mouse IgG-coated wells were included as controls. To confirm specificity of TN-C detection, aggrecan, a major cartilage proteoglycan purified from human cartilage as described [26] was tested at various concentrations in the assay as a negative control. To further confirm specificity of detection in synovial fluid, two human synovial fluids were immunodepleted of TN-C using anti-TN-C 4C8MS monoclonal antibody (Immuno-Biological Laboratories Co.) against the FNIII-B domain [27], or anti-human TN-C BC-24 (Abcam, Cambridge, MA) against the EGF domain, and then analyzed in the ELISA. Protein-G Dynabeads (Invitrogen) were used following manufacturer's protocol for immunoprecipitation (20 μg antibody; 200 μl of synovial fluid), Mouse IgG was used as a negative control in immunodepletion experiments. In order to determine spike-in recovery of TN-C, two human synovial fluids diluted to 1:100, 1:200, or 1:400 were spiked in with TN-C standard at a final concentration of 5 or 10 ng/ml and analyzed in the ELISA.

Protein was quantified using the microplate Bradford protein assay (Thermo Scientific, Rockford, IL). Cell toxicity was determined in primary cell and explant cultures by measuring lactate in the conditioned media using a lactate assay (Trinity biotech, St. Louis, MO). Prostaglandin E_2 _(PGE_2_) release was measured using a PGE_2 _ELISA (R&D Systems, Minneapolis MN). Measurement of nitrate concentrations was performed using a nitrate/nitrite colorimetric assay kit (Cayman Chemical Company, Ann Arbor, MI). Human chondrocyte conditioned media were screened using a human proinflammatory 7-plex MSD tissue culture kit (Meso Scale Discovery, Gaithersburg, Maryland). Human IL-6 and IL-8 were measured individually using MSD human cytokine assay tissue culture kits (Meso Scale Discovery). The proteoglycan content in bovine explant conditioned media was measured as sulfated glycosaminoglycan (sGAG) by a colorimetric assay with dimethylmethylene blue (Serva, Heidelberg, Germany) [28]. Proteoglycan levels in human synovial fluids were determined by the sGAG assay (Kamiya Biomedical Company, Seattle, WA) [29]. ARG-aggrecan fragments in synovial fluids were measured in an ELISA developed at Pfizer [26].

### Gene expression assays

Taqman gene expression assays were done using one-step RT-PCR reagents and Assay on Demand primer-probe sets (Applied BioSystems, Foster City, CA) following manufacturer's protocol. For analyzing bovine samples, GAPDH (Bt03210913_g1), and ADAMTS4 (Bt03224693_m1) primer/probe sets were used. For the human samples, GAPDH (Hs99999905_m1), ADAMTS4 (Hs00192708_m1), ADAMTS5 (Hs00199841_m1), and TN-C (Hs01115665_m1) primer/probe sets were used. 100 ng RNA per sample was tested in duplicates and results averaged.

### Statistical analysis

One-way Analysis of Variance (ANOVA) of log transformed values was performed for TN-C and ARG-aggrecan levels in human and rat joint fluids to test for statistical significance. Student's t-test was performed for the TN-C protein and mRNA expression studies and *in vitro *inhibition studies to test for significance. Spearman rank order was used for correlation analysis.

## Results

**Figure 1:**
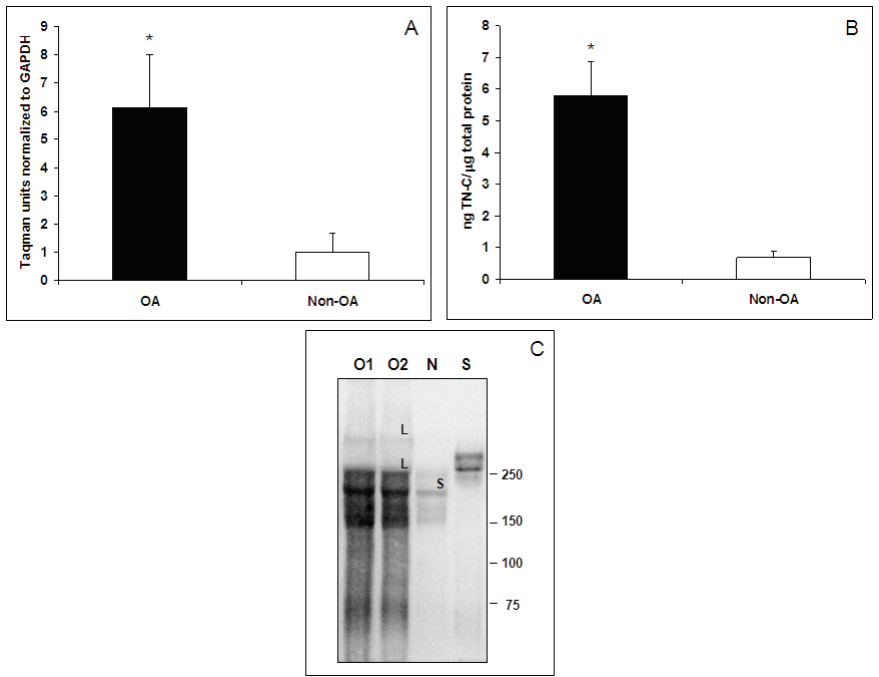
***TN-C expression in OA vs. non-OA human cartilage***. A, mRNA expression of TN-C in human cartilage expressed as Taqman units normalized to GAPDH. Non-OA has been set to 1. n = 7 donors each group; error bars indicate SD; p = 0.02. B, Protein expression of TN-C in cartilage as measured in the TN-C Large ELISA. N = 7 donors each group; error bars indicate SD; p = 0.0006. C, Representative OA and non-OA cartilage extracts (5 μg protein) separated on a 3-8% tris-acetate gel, transferred, and probed with anti-TN-C (EGF-like domain) antibody, 4F10TT that detects all forms of TN-C. Lanes are O = OA; N = Non-OA; S = standard human TN-C protein (Millipore). Bands are marked as L = large variant and S = small variant.

TN-C mRNA expression was significantly upregulated by approximately 6-fold (p = 0.02) in OA relative to non-OA cartilage (Figure 1A). An ELISA, which measures large splice variants of TN-C (TN-C Large) [30], was then used to measure TN-C protein levels. TN-C standard or samples plated on PBS- or mouse IgG coated wells did not produce any optical density (OD) values in the ELISA confirming specific binding of TN-C to 19C4MS-coated plates. Aggrecan tested as a negative control did not produce signal further confirming the specificity of detection. OA cartilage (n = 7) had a mean of 5.79 ng TN-C per μg total protein, which was significantly higher (approximately 8-fold; p = 0.0006) than the levels in non-OA cartilage (n = 7) which gave a mean of 0.69 ng per μg total protein (Figure 1B). In the Western immunoblot analyses of representative cartilage extracts, we also observed increased TN-C levels in OA cartilage extracts (Figure 1C). Two large variants of 350 and 240 kD molecular weight, and a small variant at 210 kD were observed in OA cartilage. The non-OA cartilage extracts had only the 240 kD large variant and the small 210 kD variant.

Purified TN-C protein (Millipore) consisting of large variants was tested for endotoxin levels using the Endosafe-PTS that utilizes existing FDA-licensed LAL (Limulus amaebocyte lysate) formulations loaded into a test cartridge. The level measured prior to endotoxin removal was 8.0 endotoxin units (EU)/mg protein. After passing the protein through detoxigel endotoxin removal columns, the levels dropped to < 0.05 EU/mg protein in the Endosafe-PTS assay. When human primary chondrocytes were treated with varying concentrations of TN-C or LPS (1-10 μg/ml TN-C; 0.1-1000 ng/ml LPS) and conditioned media samples screened using the proinflammatory 7-plex MSD kit (for IFN-γ, IL-1β, IL-6, IL-8, IL-10, IL-12p70, and TNFα), only IL-6 and IL-8 were detected and found to be significantly induced by TN-C or LPS treatments (data not shown). Individual IL-6 and IL-8 MSD tissue culture kits were used for further confirmation.

**Figure 2:**
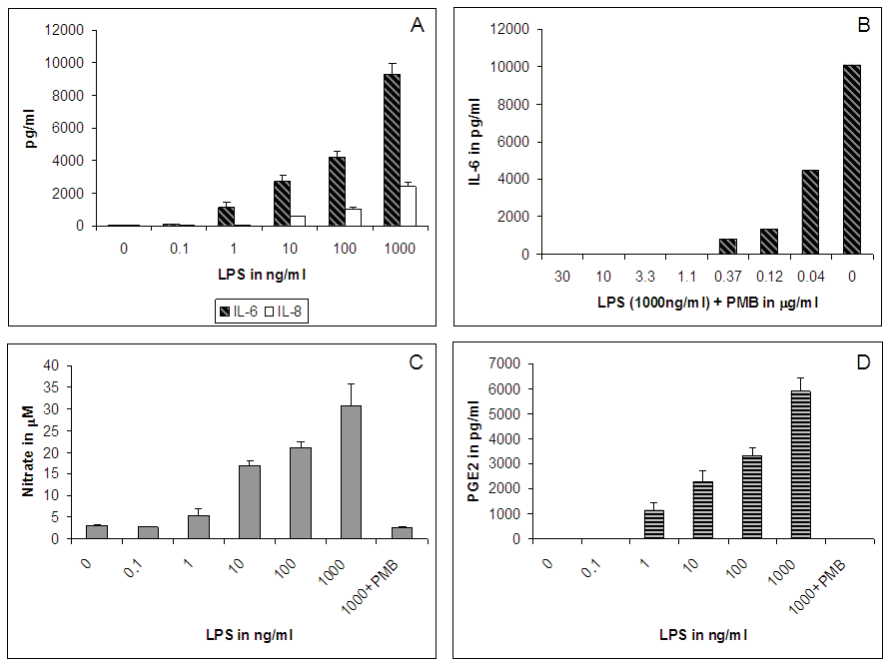
***LPS induction of primary human chondrocytes***. Release of A, IL-6 and IL-8; C, nitrate; and D, PGE_2 _from cultured human chondrocytes treated with 0-1000 ng/ml LPS for 48 hours. B, Dose dependent inhibition of LPS (1000 ng/ml) induced IL-6 release by polymyxin-B (PMB). PMB was added at 3 μg/ml in C and D. The error bars indicate SD of averaged treatment results (duplicate wells) from 3 donors.

LPS tested at 0.1 to 1000 ng/ml induced IL-6 and IL-8 release from human primary chondrocytes dose-dependently resulting in 20-170 fold induction of IL-6 at 1 to 1000 ng/ml LPS, and 15-60 fold induction of IL-8 at 10 to 1000 ng/ml LPS. No significant increase over control was observed for IL-6 at 0.1 ng/ml LPS; and for IL-8 at 0.1 and 1 ng/ml LPS (Figure 2A). IL-6 was followed up in further experiments with chondrocytes and explants. A dose dependent inhibition of IL-6 release by PMB was observed; 100% inhibition of IL-6 release was seen at 1 μg/ml PMB (Figure 2B). 1000 ng/ml LPS that was pre-incubated with 3 μg/ml PMB served as a negative control for LPS treatment in further experiments. Nitrate release was induced 2-10 fold (Figure 2C) and PGE_2 _release increased 350-1750 fold in a dose dependent fashion when tested at 1 to 1000 ng/ml LPS. There was no significant change in the release of nitrate and PGE_2 _at 0.1 ng/ml LPS (Figure 2D).

**Figure 3:**
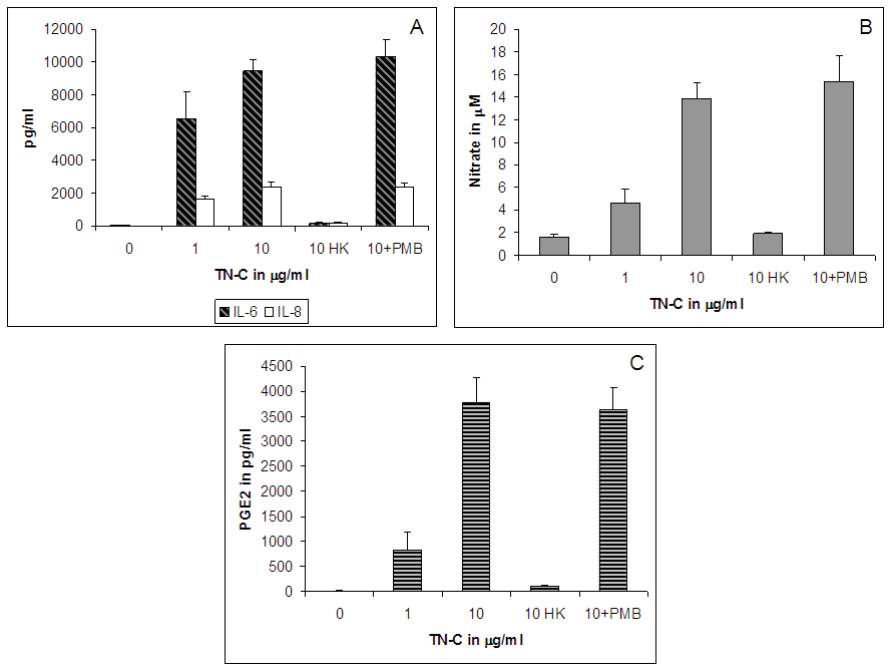
***TN-C treatment of primary human chondrocytes***. Release of A, IL-6 and IL-8; B, nitrate; C, PGE_2 _from cultured human chondrocytes treated with 0-10 μg/ml TN-C for 48 hours. HK- heat killed. 10 μg/ml heat killed TN-C served as a negative control. The error bars indicate SD of averaged treatment results (duplicate wells) from 3 donors.

Addition of TN-C protein (1 or 10 μg/ml) to human chondrocyte cultures induced IL-6, IL-8, PGE_2 _and nitrate in a dose dependent manner (Figure 3). TN-C treatment resulted in an approximately 160- and 230-fold increase in IL-6 release; 80- and 120-fold increase in IL-8 release (Figure 3A); 3- and 8-fold increase in nitrate release (Figure 3B); and 130- and 600-fold increase in PGE_2 _(Figure 3C) release at 1 and 10 μg/ml, respectively. TN-C at 10 μg/ml pretreated with 3 μg/ml PMB did not show any reduction in IL-6, IL-8, nitrate and PGE_2 _release. 10 μg/ml heat killed TN-C served as a negative control in this experiment and did not show induction of IL-6, IL-8, PGE_2_, or nitrate (Figure 3). The results from PMB treated TN-C and heat killed TN-C confirmed that the effects observed were endotoxin independent.

**Figure 4:**
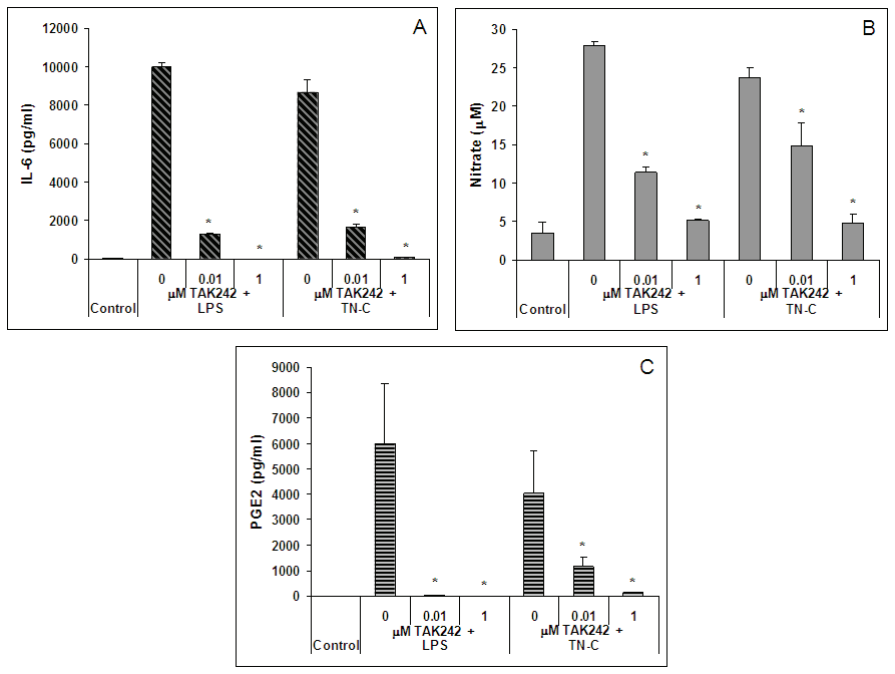
***Effect of TAK242 on LPS or TN-C induced changes in primary human chondrocytes***. Human chondrocytes were induced with 1000 ng/ml LPS or 10 μg/ml TN-C for 48 hours. TAK242 was present 2 hours prior to induction and during the induction period. TAK242 effects on TN-C or LPS induced A, IL-6; B, nitrate; and C, PGE_2 _release. The error bars indicate SD of averaged treatments (duplicate wells) from 3 donors. * indicates p < 0.05.

Induced IL-6, PGE_2_, and nitrate release with 1000 ng/ml LPS or 10 μg/ml TN-C treatment was dose-dependently inhibited by TAK242. TAK242 at 1 μM resulted in complete inhibition of LPS or TN-C induced release of IL-6, PGE_2 _and nitrate (Figure 4). Lactate concentrations in the media (≥ 85% lactate compared to control wells) confirmed that inductions and inhibitor treatments were tolerated by the cells at the concentrations used.

**Figure 5:**
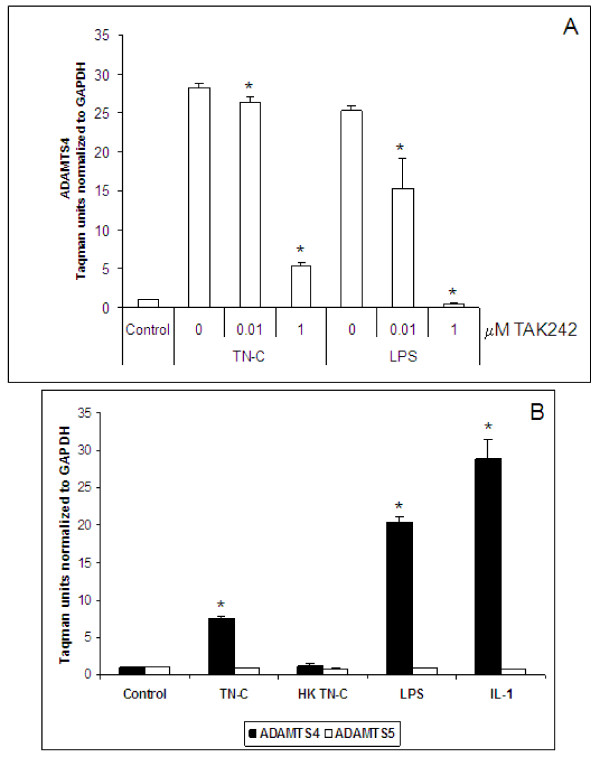
***TN-C or LPS treatment and ADAMTS expression in primary chondrocytes***. A, Bovine chondrocytes were induced with 10 μg/ml TN-C or 1000 ng/ml LPS for 48 hours with or without TAK242. TAK242 was present 2 hours prior to induction and during the induction period. B, Human chondrocytes were induced with 10 μg/ml TN-C, 1000 ng/ml LPS, or 5 ng/ml IL-1β for 48 hours. Cells were lysed, RNA prepared and taqman assays performed with 50 ng RNA/well. HK = heat killed. ADAMTS = A Disintegrin And Metalloproteinase with Thrombospondin Motifs. The error bars indicate SD of treatments done in triplicates, each RNA sample from treatments/wells was tested in duplicates in the Taqman assay and results averaged. * p < 0.05.

ADAMTS4 mRNA expression in bovine chondrocytes was up regulated 28- and 25-fold when treated with 10 μg/ml TN-C and 1000 ng/ml LPS, respectively. Upregulation of ADAMTS4 by TN-C or LPS was dose-dependently suppressed by TAK242 (Figure 5A). Similarly, ADAMTS4 mRNA expression was upregulated 8- and 20-fold in human primary chondrocytes when treated with 10 μg/ml TN-C and 1000 ng/ml LPS, respectively. IL-1β at 5 ng/ml that was used as a positive control resulted in 29-fold up regulation of ADAMTS4 in human chondrocytes. (Figure 5B). In contrast to ADAMTS4, ADAMTS5 did not show any significant changes with TN-C, LPS or IL-1β treatment. Heat killed TN-C at 10 μg/ml and PMB treated LPS at 1 μg/ml served as negative controls and did not cause significant upregulation of ADAMTS4.

**Figure 6:**
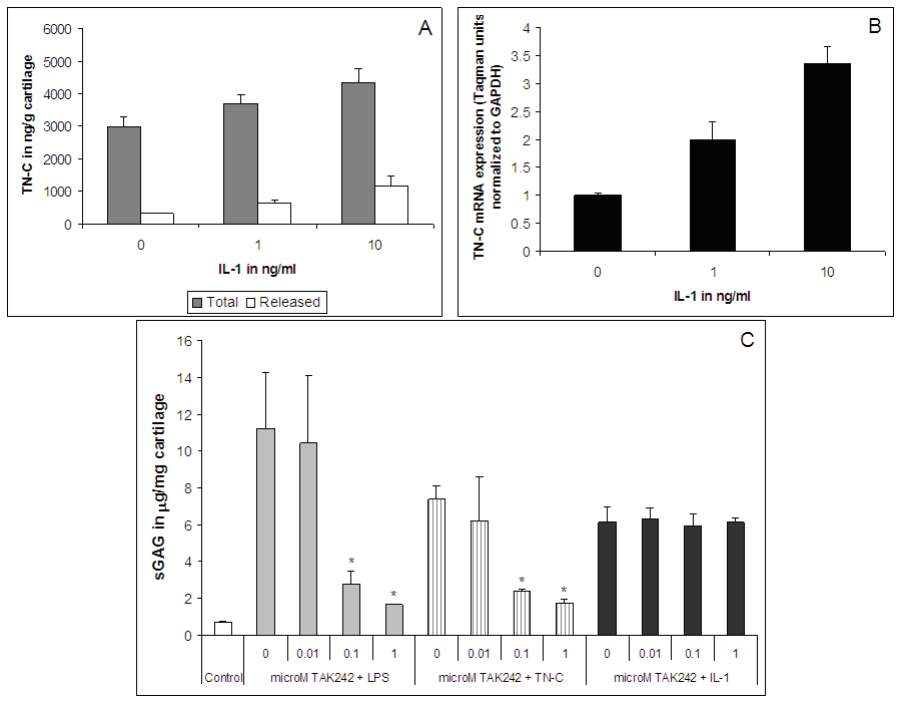
***IL-1α induced TN-C synthesis and LPS/TN-C dependent proteoglycan loss in bovine explant cultures***. Bovine explants (3 discs/well) were induced with IL-1α (0, 1, 10 ng/ml) for 48 hours and A, cartilage extracts and conditioned media analyzed in the TN-C Large ELISA. The error bars indicate SD of treatments done in triplicates; B, cartilage RNA prepared and TN-C taqman analysis performed with 50 ng RNA/well. The error bars indicate SD of treatments done in triplicates; each RNA sample from treatments/wells was tested in duplicates in the Taqman assay and results averaged. * p < 0.05. C, Bovine explants (3 discs/well) were induced with 1000 ng/ml LPS, 10 μg/ml TN-C or 5 ng/ml IL-1α in the presence or absence of TAK242 (0, 0.01, 0.1, or 1 μM) for 48 hours and the conditioned media analyzed for sGAG content. * p < 0.05.

IL-1α added at 0, 1, and 10 ng/ml to bovine explant cultures increased TN-C protein in the cartilage and also stimulated the release of TN-C into the conditioned media in a dose-dependent manner (Figure 6A). The increase in TN-C protein levels correlated with mRNA expression in the cartilage (Figure 6B). Proteoglycan loss was induced as measured by sGAG release into the conditioned media of bovine explants following 10 μg/ml TN-C or 1000 ng/ml LPS treatment, which was similar to the loss due to 5 ng/ml IL-1α induction (Figure 6C). IL-1α resulted in approximately 20% loss of sGAG from bovine cartilage in 48 hours. TN-C showed a similar percentage release, whereas, the release with LPS was slightly higher at approximately 30% loss. TAK242 (at 0.1 and 1 μM) dose dependently reversed the loss of proteoglycan due to TN-C and LPS treatments, but did not affect IL-1α induced proteoglycan release (Figure 6C).

**Figure 7:**
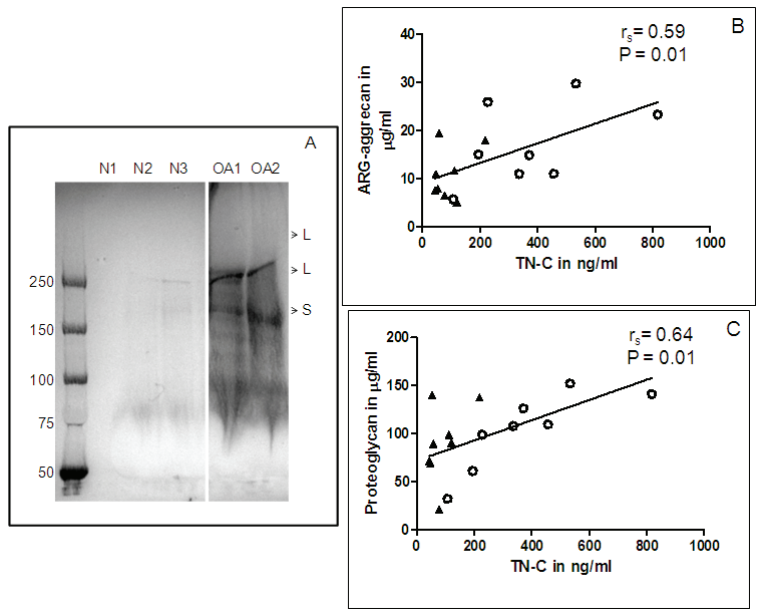
***TN-C in human synovial fluids and its correlation to proteoglycan release***. A, Western blot analysis of human synovial fluid. Representative samples (15 μl each) were treated with 10 U of hyaluronidase overnight at RT, separated on a 3-8% tris-acetate gel, transferred, and probed with anti-TN-C (EGF-like domain) antibody, 4F10TT that detects all forms of TN-C. Lanes are N = synovial fluid of non-OA reference subjects; O = synovial fluid of OA patients. Bands are marked as L for large variants and S for small variants. B, Correlation analysis of TN-C *vs*. ARG-aggrecan in human synovial fluid. C, Correlation analysis of TN-C *vs*. total proteoglycan in human synovial fluid. For both B and C, N = 8 each group; triangles represent non-OA reference subjects and circles represent OA patients.

Human synovial fluids depleted of TN-C with anti-TN-C antibodies prior to testing showed 100% loss of signal in the ELISA confirming the specificity of detection in synovial fluids. The mean spike-in recovery of TN-C at three different dilutions tested was 89% with a range of 78-97%. TN-C level measured in human OA synovial fluids (n = 8) gave a mean of 380 ng/ml (range: 107-817 ng/ml), whereas, the mean of TN-C in the reference synovial fluids was 90 ng/ml (range: 44-218 ng/ml) giving a significant 4.2-fold higher release in the OA group as compared to the healthy reference controls (p = 0.001). Figure 7A shows the results of Western immunoblot analysis of representative OA and non-OA synovial fluid samples using anti-TN-C antibody. As in the OA cartilage extract, 350 kD and 240 kD large TN-C variants and the 210 kD small variant were present in the OA synovial fluids. TN-C was present at insignificant levels in non-OA reference fluids. Our Western immunoblot analysis results correlated with the TN-C bands reported earlier in OA synovial fluids [14]. Upregulation of TN-C mRNA and protein in the cartilage correlated significantly with a simultaneous increase in the synovial fluid; the correlation analysis of these factors tested in the same OA patients have been summarized in Table 1.

**Table 1:** Correlation analysis of TN-C protein and mRNA in cartilage *vs*. TN-C protein in synovial fluid of OA patients

	TN-C protein in cartilage	TN-C protein in SF
TN-C mRNA in cartilage	r_s _= 0.9286 P = 0.0005	r_s _= 0.8080 P = 0.0059

TN-C protein in cartilage		r_s _= 0.8696 P = 0.0022

Note: TN-C in the synovial was measured in the ELISA that quantifies only large variants of TN-C. SF: synovial fluid; N = 7 each from the same individuals; Spearman rank order was used for correlation analysis; P indicates p values.

**Figure 8:**
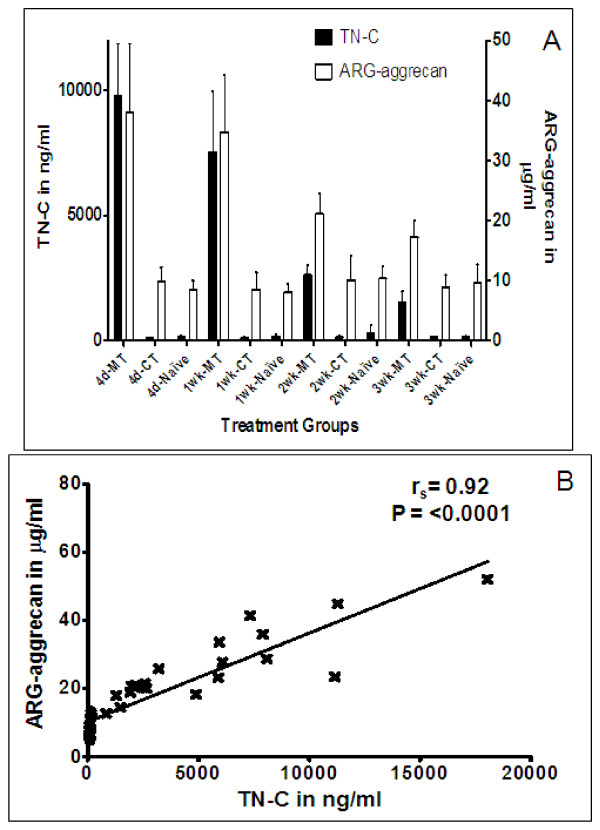
***TN-C vs. ARG-aggrecan release in the rat meniscal tear model***. A, Time course of TN-C *vs*. ARG-aggrecan release. Rats were euthanized at different times after surgery (4 days, 1 wk, 2 wks, and 3 wks; n = 10 rats/time point). Five naïve rats (10 joints) per time point were included. For each time point, 10 synovial fluid lavage samples from the right meniscal tear (MT), left contra-lateral (CT), and naïve (left +right knees) joints were tested for TN-C and ARG-aggrecan and values corrected for dilution using serum urea levels. Average values are shown with the error bars indicating SD. B, Correlation analysis of TN-C *vs*. ARG-aggrecan release in the rat meniscal tear model. TN-C and ARG-aggrecan data from the first five animals (n = 5 MT right knees and 5 CT left knees) of each time point (4 days, 1 wk, 2 wks, and 3 wks) were plotted in a correlation plot. Circles represent values from the CT knees and crosses represent values from the MT knees (n = 40 CT and 40 MT).

A trend towards correlation was observed when TN-C levels were correlated to aggrecanase generated ARG-aggrecan or total proteoglycan in human synovial fluid samples tested (Figure 7B &7C). In the rat meniscal tear model, there was a significant 107-fold increase in TN-C release at 4 days in surgery knees compared to no surgery contralateral left controls or the knees of naïve animals, the fold increase dropped to 77-, 20- and 12-fold increase at 1-, 2- and 3-wks after joint instability induction, respectively. The trend of TN-C release into the synovial fluids followed the release of ARG-aggrecan in these animals; ARG-aggrecan of rat joint fluids showed a significant 4 fold increase in the unstable right knees at 4 days and 1 wk after surgery as compared to un-operated contra-lateral left knees or naïve animals (left and right knees), the fold increase dropped gradually at 2 and 3 wks post surgery but was significantly higher than the controls (Figure 8A). There was a very significant correlation (r_s _= 0.92 and p = < 0.0001) when the TN-C levels in these samples (n = 40; fluids from left contra lateral and right surgery knees of 5 animals per time point) were correlated to ARG-aggrecan levels (Figure 8B).

## Discussion

In the current study, we found a concomitant upregulation of TN-C mRNA and protein in the cartilage along with increased TN-C in the synovial fluid of OA patients. We have demonstrated a novel role for increased TN-C levels in the OA joint in promoting proteoglycan loss in addition to mediating inflammatory signals, which is supported by a correlation between TN-C levels in the knee synovial fluid and proteoglycan loss from the articular cartilage in human and rat joints.

In musculoskeletal tissues, the factors regulating the expression of TN-C are IL-1β [31], tumor necrosis factor-α (TNFα) [31], transforming growth factor-β (TGFβ) [32], and basic fibroblast growth factor (bFGF) [33], all of which are present at increased levels in the joints of patients with OA compared with those of normal patients [34]. A range of TN-C variants with mass from 350 to 210 kD are generated by alternative splicing of FN (III) A-D repeats of TN-C RNA [35]. Studies have shown that TN-C is localized in articular cartilage from OA patients at the extracellular matrix underneath the surface and pericellular compartment of the chondrocytes [10,11]. Chondrocytes also produce both large and small variants during embryogenesis and development and provide a cellular source of TN-C in the synovial fluid [2,9]. We observed that both large (350 and 240 kD) and small (210 kD) variants were abundant in OA cartilage extracts and synovial fluids when compared to non-OA samples. Our findings agree with an earlier report where all TN-C variants were found to increase in the synovial fluid with advancing stages of cartilage degradation [14]. Western blotting revealed degraded fragments of TN-C of molecular weight lower than 200 kD in OA cartilage and synovial fluid (Figure 1C &7A) that could be MMP generated [36]. The large variants are known to be more susceptible to MMP cleavage, such as MMP-2 and MMP-7, than the small variant [36]. This is evidenced by the relatively lower intensity of the 350 kD large variant in OA cartilage and synovial fluids. ILα increased TN-C levels in cartilage in culture as well as its release into the conditioned media indicating enhanced synthesis of TN-C by chondrocytes in response to inflammatory stimuli, this is in agreement with an earlier finding on ILβ induced TN-C in human cartilage [37].

LPS and other microbial components initiate signal transduction through TLR4, resulting in the release of inflammatory cytokines. TLR4 also binds to matrix components that include heparin sulfate, fibronectin, biglycan, and hyaluronan [38]. TN-C was recently added to the list of endogenous activators of TLR4 [19]. Signal transduction through TLR4 leads to the activation of transcription factors, and in turn controls the expression of proinflammatory cytokines, chemokines, and MMPs [39,40]. Expression of TLR4 in human OA chondrocytes and cartilage in our study was confirmed by qPCR (data not shown). Expression of TLR4 and its adaptors have been reported also in human OA synovium [41]. Synovial tissue from OA stifle dog joints that underwent cranial cruciate ligament transaction was shown to have significantly higher TLR4 gene and protein expression as compared to the non-OA contralateral joints [42].

TN-C levels measured in the eight human synovial fluids (based on data availability for both TN-C and ARG-aggrecan levels) included in the study ranged from 0.11-0.82 μg/ml. However, we have measured levels up to 5 μg/ml in several other human OA synovial fluids tested. TN-C in dog synovial fluid after anterior cruciate ligament transection (ACLT) also went up to 5 μg/ml similar to human OA samples (data not shown). A dose of 1-10 μg/ml TN-C was used in our *in vitro *experiments to keep the treatment level close to physiological levels in the joint under diseased conditions. TN-C induced inflammatory mediators including IL-6, IL-8, nitrate and PGE2 in the cartilage *in vitro *in a fashion similar to LPS in our study. TAK242, the TLR4 specific small molecule inhibitor binds strongly and specifically to TLR4. It inhibits TLR4 signaling by binding to Cys747 in the intracellular domain of TLR4 [43]. We used TAK242 to confirm that the role of TN-C in inducing inflammatory mediators in articular cartilage is TLR4-dependent. Our results agree with the earlier findings in human macrophages and fibroblasts from synovia of RA patients [19].

Loss of ECM from articular cartilage is a central event that leads to joint destruction in arthritic diseases. Aggrecan is a major component of the ECM responsible for weight bearing, and an important factor in the retention of collagen within matrix [44]. Aggrecanases are responsible for degrading aggrecan in articular cartilage [45]. TN-C upregulated ADAMTS4 expression in chondrocytes *in vitro via *TLR4 signaling that reflected in increased loss of sGAG from the cartilage matrix. We tested the effect of added LPS (1000 ng/ml) or TN-C (10 μg/ml) for 48 hrs on aggrecan mRNA expression in human primary chondrocytes using Taqman assays and found no significant regulation in aggrecan expression with treatment. TN-C or LPS treatment at the above concentrations and duration also did not result in any significant change in the proliferation rate of the primary cells tested by the bromodeoxyuridine incorporation method (data not shown). Proteoglycan loss measured as sGAG might indicate regeneration of cartilage, however, lack of TN-C or LPS induced changes in the proliferation rate and in aggrecan expression suggests that the enhanced release of sGAG results from matrix degradation; this is supported by the observed upregulation of ADAMTS4 in response to TN-C or LPS treatment. ADAMTS5 did not respond to induction with LPS, TN-C or IL-1β in our primary chondrocyte induction experiments, consistent with earlier reports on induced gene expression in cartilage [46,47]. However, TN-C has been shown to be upstream in the regulation of several MMPs in synovial fibroblasts [48].

Increased levels of TN-C in the joint fluid significantly correlated with cartilage TN-C mRNA and protein levels in OA patients (Table 1). Similarly, correlating with enhanced release of TN-C from rat joints due to surgical induction of OA, we observed a slight but statistically significant upregulation of TN-C mRNA in the transcriptional profiling studies of cartilage from the knees of rats that underwent meniscal tear as compared to cartilage from the contralateral knees (34% upregulation; p = 0.009), 2 weeks post surgery. Our findings on correlation between TN-C levels and proteoglycan loss in human and rat joints are consistent with a recent report showing decreased proteoglycan staining accompanied by increased tenascin deposition in human cartilage with OA lesions [49]. The correlation between TN-C and aggrecan loss could result from two different roles of TN-C: 1) TLR4 dependent TN-C induction of matrix degradation whereby TN-C regulates the expression metalloproteases and 2) Loss of TN-C along with degraded fragments of aggrecan resulting from aggrecanase activity in diseased cartilage as TN-C binds to the alternatively spliced G3 domain of aggrecan [50]. Our results suggest an important role for TLR4 in the pathological process initiated by elevated TN-C in the diseased joints; testing TAK242 in the rat meniscal tear model of OA might provide additional information.

Increased intensity of TN-C staining has been observed in areas of damaged human OA cartilage compared with normal cartilage [51], and a strong correlation between joint fluid TN-C levels and OA severity has also been reported [14]. A role for TN-C in the assembly of the chondrocyte matrix has been reported [52]. Treatment of human articular chondrocytes with TN-C was also shown to accelerate chondrocyte proliferation and play a role in cartilage repair [49]. These findings suggest involvement of TN-C in tissue remodeling that occurs in conjunction with degeneration and repair, which is further emphasized by the delay in articular cartilage repair observed for TN-C-deficient mice [53]. Indeed, we observed a pronounced increase in TN-C release into the joint fluid immediately after surgery in the rat model of OA/joint injury (Figure 8); TN-C levels decreased with time after surgery, indicating the transient expression of TN-C during the repair process. Similar patterns of TN-C release with a pronounced increase immediately after injury/disease onset that gradually reduced over time was observed when human knee synovial fluids from acute cruciate ligament injury, meniscal injury, and acute inflammatory arthritis patients were tested (data not shown).

We hypothesize that TN-C which reappears to attempt repair and remodeling in the OA joint could induce cytokines, inflammatory mediators, and matrix degrading enzymes and result in propagation of inflammation and matrix degradation through TLR4 signaling. TLR4 expression has been shown to increase in human OA cartilage lesions, and TLR4 ligands strongly induce catabolic responses in human chondrocytes including production of MMPs 1, 3, and 13 and of nitric oxide (NO), and PGE2, as well as a significant increase in the release of proteoglycan and type II collagen degraded products; treatment with TLR4 ligands led to phosphorylation of p38, ERK, and JNK, and activation of NFκB [54]. The active domain of TN-C that activates cells in the joint has been mapped to the fibrinogen-like globe of the molecule [19]. Stimulation of cytokines in synovial fibroblasts *via *activation of TLR4 was MyD88-dependent [19]; MyD88 knockdown in human chondrocytes inhibited IL-1 induced expression of metalloproteases [55] suggesting MyD88 as a potential target in addition to TLR4 to intervene cartilage degradation. The rat meniscal tear model of OA and the TN-C time course release pattern explored in this study could serve to evaluate TLR4 or MyD88 inhibitors, and in turn confirm the role of TLR4 signaling and TN-C in OA progression. Further studies to explore the signaling pathway of TN-C induced TLR4 in chondrocytes that leads to inflammation and cartilage matrix degradation are warranted.

## Conclusions

TN-C mRNA and protein are upregulated in articular cartilage along with an increase in TN-C levels in the synovial fluid of OA patients. TN-C is inducible in primary chondrocytes by the inflammatory cytokine, IL-1; it is capable of stimulating further inflammatory mediators and promoting proteoglycan degradation in articular cartilage *in vitro*. TN-C release into the joint fluid correlates with aggrecan loss in human and rat OA joints. *De-novo *expression of TN-C appears to be a reliable marker of joint injury/disease.

## List of Abbreviations

TN-C: Tenascin-C; OA: osteoarthritis; IL-1: Interleukin-1; ECM: extracellular matrix; LPS: lipopolysaccharide; DAMP: damage-associated molecular pattern; EGF: epidermal growth factor; FN: fibronectin; RA: rheumatoid arthritis; TLR: Toll-like receptor; MMP: metalloprotease; PMB: polymyxin-B; Ref: knee-healthy reference subjects; PGE_2_: Prostaglandin E_2_; sGAG: sulfated glycosaminoglycan; LAL: Limulus amaebocyte lysate; EU: endotoxin units; IFN: interferon; TNFα: Tumor necrosis factor-α; bFGF: basic fibroblast growth factor; TGFβ: transforming growth factor-β; NO: nitric oxide; ERK: extracellular signal-regulated kinase; JNK: c-Jun N-terminal kinase; NFκB: nuclear factor kappa-light-chain-enhancer of activated B cells; SF: synovial fluid; CT: contralateral; MT: meniscal.

## Competing interests

LP has no competing interests. WS, SSG, EAM, CRF, and PSC are current or past employees of Pfizer and hold company stocks/stock options.

## Authors' contributions

LP was primarily responsible for the experimental set up, data acquisition and analysis, and contributed to manuscript preparation. WS performed Western blot analysis, ARG-aggrecan ELISA, and some human chondrocyte experiments. SSG was responsible for the rat model and related samples. EAM contributed to the concept of the study. CRF provided input on the experimental design and approved the manuscript. PSC was responsible for the conception and design of the study, data acquisition, analysis and interpretation, and manuscript design and preparation. All authors read and approved the final manuscript.

## Pre-publication history

The pre-publication history for this paper can be accessed here:

http://www.biomedcentral.com/1471-2474/12/164/prepub
